# A Rare Case of Pyelonephritis With Methicillin-Resistant Staphylococcus aureus (MRSA) Bacteremia Complicated by Renal Vein Thrombosis

**DOI:** 10.7759/cureus.76832

**Published:** 2025-01-02

**Authors:** Usamah Al-Anbagi, Amro A Abdelrahman, Suha S Al Hassan, Nafisa E Mohammed, Abdulqadir J Nashwan, Aram Salehi

**Affiliations:** 1 Internal Medicine, Hamad Medical Corporation, Doha, QAT; 2 Medical Education, Hamad Medical Corporation, Doha, QAT; 3 Nursing and Midwifery Research, Hamad Medical Corporation, Doha, QAT

**Keywords:** antibiotic, anticoagulation, bacteremia, methicillin-resistant staphylococcus aureus (mrsa), pyelonephritis, renal vein thrombosis (rvt), urinary tract infection (uti)

## Abstract

Pyelonephritis is a significant urinary tract infection (UTI) that can lead to serious complications, including bacteremia and renal vein thrombosis (RVT). This report presents a case of pyelonephritis caused by methicillin-resistant *Staphylococcus aureus* (MRSA), which progressed to RVT. We report a case of a 44-year-old male patient who presented with dysuria, increased urinary frequency, and urine retention over the past three days. His condition deteriorated, leading to reduced urine output and acute urinary retention. Notably, the patient did not report pyuria, hematuria, or fever, and there was no history of nephrolithiasis or recent urogenital procedures. Blood and urine cultures confirmed the presence of MRSA. A contrast-enhanced CT scan of the abdomen revealed left pyelonephritis complicated by abscess formation in both the left kidney and the prostate, as well as left RVT. This case highlights the potential complications of pyelonephritis, such as RVT, and underscores the importance of early diagnosis and intervention. Clinicians should maintain a high vigilance for RVT in patients with pyelonephritis and bacteremia, as timely management can significantly improve patient outcomes.

## Introduction

Renal vein thrombosis (RVT) is a condition characterized by the formation of a thrombus in the renal veins or their branches, leading to impaired venous drainage of the kidneys. Although relatively rare, RVT occurs more commonly in specific populations, including adults with nephrotic syndrome, neonates experiencing dehydration or volume contraction, and individuals with inherited thrombophilia or hypercoagulable states [1-2]. The clinical presentation of RVT can vary widely. Acute cases may manifest with symptoms such as flank pain, gross hematuria, renal swelling, and acute kidney injury [2]. However, a significant number of cases remain asymptomatic and are only identified incidentally during imaging studies performed for other reasons or when complications such as pulmonary embolism or progressive renal dysfunction arise.

While the potential severity of RVT is well-recognized, its association with pyelonephritis, a bacterial infection of the renal parenchyma and urinary tract, is notably rare and underexplored. Pyelonephritis primarily causes systemic symptoms such as fever, flank pain, and urinary abnormalities but is not commonly linked to thrombotic complications like RVT [3]. Several reports have highlighted the clinical significance of this rare association, suggesting a need for greater awareness and investigation into potential underlying mechanisms that may connect these conditions [3-5].

In this report, we describe the case of a 44-year-old man with poorly controlled diabetes mellitus who developed RVT secondary to acute pyelonephritis (APN) caused by methicillin-resistant *Staphylococcus aureus* (MRSA). This case illustrates the concept of "thrombo-inflammation," where the inflammatory response to infection can contribute to developing thrombosis.

## Case presentation

A 44-year-old male patient presented to the emergency department with acute urinary retention for one day. His symptoms had begun three days earlier with increased urinary frequency, followed by a gradual decrease in urine output until he was completely unable to pass urine. He denied abdominal pain, pyuria, hesitancy, hematuria, or urinary discharge. He also reported no fever or previous urological conditions. His past medical history included diabetes mellitus, for which he was taking metformin. A review of the systems was unremarkable.

On clinical examination, his temperature was 37.2°C, his heart rate was 101 beats per minute, his respiratory rate was 18 breaths per minute, his blood pressure was 131/91 mmHg, and his oxygen saturation was 93% on room air. He appeared pale with reduced skin turgor. Abdominal examination revealed suprapubic tenderness, while the examination of other systems was unremarkable. A Foley catheter was inserted.

Blood tests revealed a white blood cell count (WBC) of 11.6 x 10⁹/L, a hemoglobin level of 10.0 g/dL, a creatinine level of 138 µmol/L, a sodium level of 124 mmol/L, and C-reactive protein (CRP) of 358.8 mg/L. Urinalysis showed elevated levels of WBC (2,694) and red blood cell (RBC) (56), and the urine dipstick was positive for nitrates. A urine culture was also sent for analysis (Table 1).

**Table 1:** Laboratory investigations HbA1c: glycated hemoglobin; AST: aspartate aminotransferase; ALT: alanine aminotransferase; CRP: C-reactive protein; TSH: thyroid-stimulating hormone; FT3: free triiodothyronine; ANA: antinuclear antibody profile; INR: international normalized ratio

Parameters	On admission	^3rd^ day	On discharge	Reference values
Total leukocytes	11.6	8.9	5.8	(6.2 x 10^3^/uL)
Hemoglobin (gm/dL)	10.4	8.1	9.8	(13-17 gm/dL)
Platelet (x10^3^/uL)	266	480	430	(150-410 x10^3^/uL)
Serum potassium K (mmol/L)	5.5	3.7	3.7	(3.5-5.3)
Serum sodium (mmol/L)	120	133	138	(133-146)
Serum calcium (mmol/L)	2.38	-	-	(2.2-2.6)
Serum magnesium (mmol/L)	0.84	-	-	(0.7-1)
Serum urea (mmol/L)	14.2	3.1	4.8	(2.5-7.8)
Serum creatinine (umol/L)	167	101	157	(62-106)
HbA1c	>12	-	-	<6%
Serum albumin (gm/L)	25	-	17	(35-50)
Serum total protein (gm/L)	72	-	56	(60-80)
AST (IU/L)	17	-	14	(0-41)
ALT (IU/L)	46	-	27	(0-41)
Alkaline phosphatase (U/L)	311	-	435	(40–129)
Serum total bilirubin (mg/dl)	13	-	8	(0-21)
Serum chloride (mmol/L)	82	104	102	(95-108)
CRP ( mg/L)	358	36	3.7	(0-5)
TSH (mIU/L)	0.4	-	-	(0.3-4.2)
FT3 (pmol/L)	18.7	-	-	(11-23.3)
Lupus anticoagulant	Not detected	-	-	Not detected
Protein C activity	65.9 %	-	-	65-140%
Protein S activity	110 %	-	-	72-126%
Antithrombin activity	91.7	-	-	79.4-130%
Anticardiolipin	Negative	-	-	Negative
Factor V Leiden variant	Not detected	-	-	Not detected
ANA profile	Negative	-	-	Negative
INR	1.3	1.5	1	<1.1
Prothrombin time	14.7	16.3	11.7	(9.5-12.5 second)

**Figure 1:**
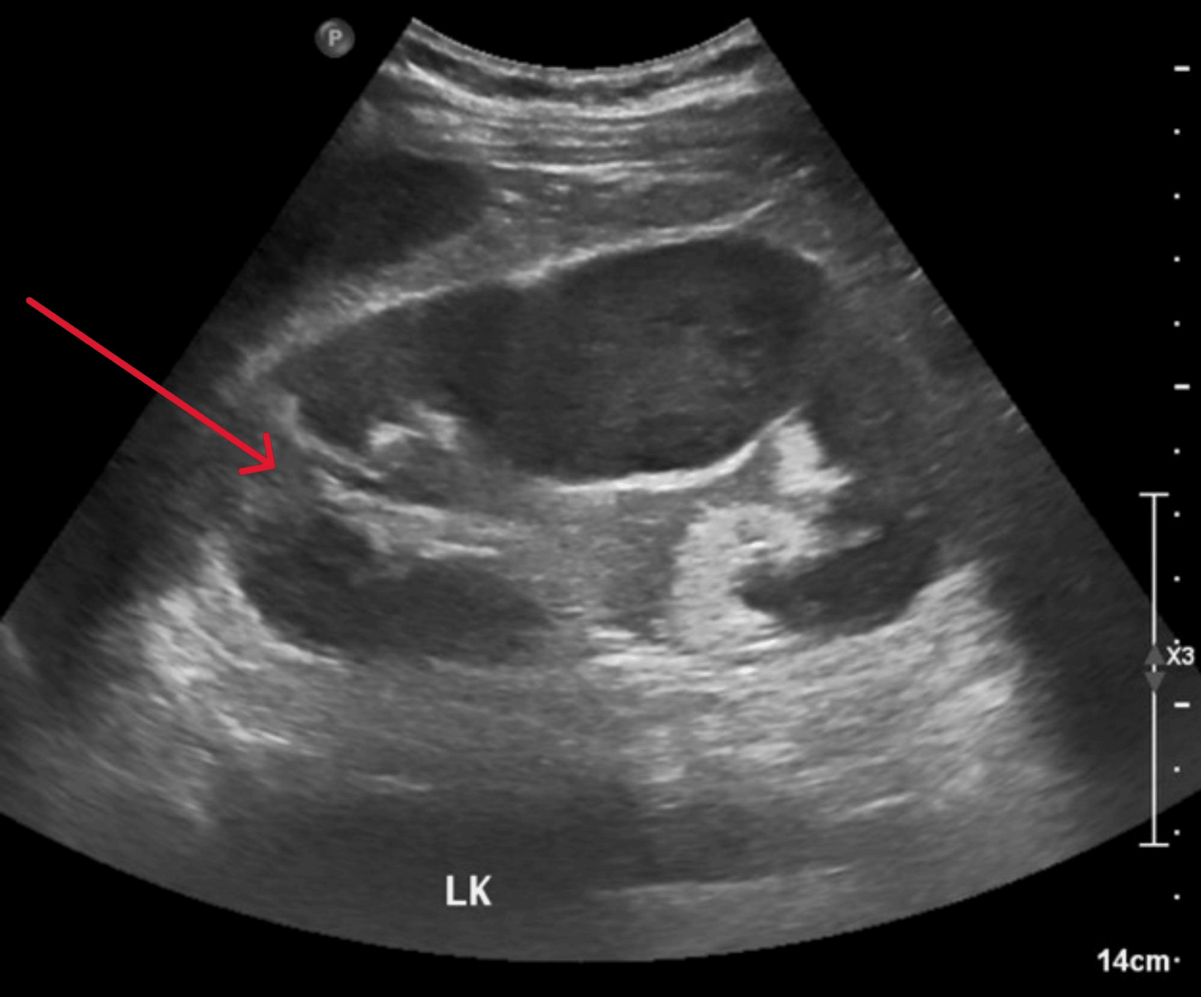
Kidney ultrasound revealed mild renal pelvic dilatation with internal echogenic debris (red arrow)

Abdominal ultrasound revealed findings suggestive of left-sided pyelonephritis (Figure 1). The patient was started on insulin, intravenous fluids, and empirical antibiotics (piperacillin/tazobactam) and was admitted for further management.

**Figure 2:**
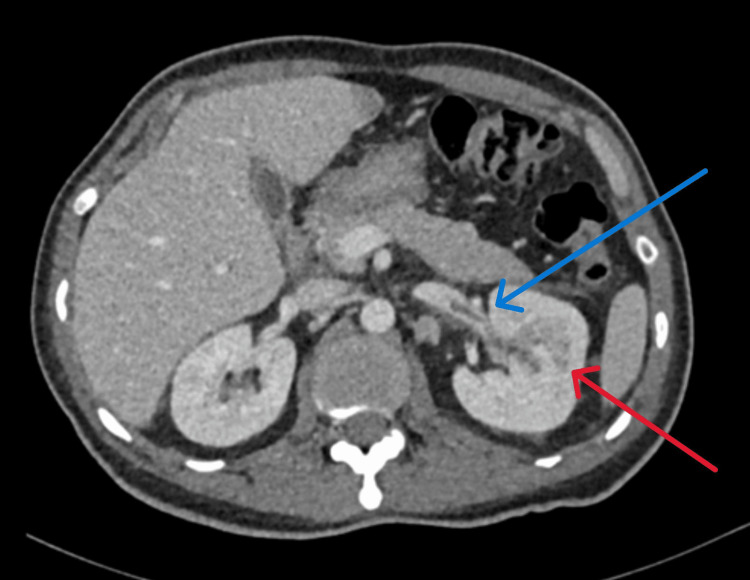
Computerized tomography abdomen (axial view) with contrast demonstrating left renal vein thrombosis (blue arrow) and pyelonephritis (red arrow)

The following day, a urologist examined the patient and noted that his prostate was enlarged upon rectal examination. To rule out a prostatic abscess, a CT scan was requested. The scan showed a multiloculated lesion in the left kidney, indicating pyelonephritis, with a renal abscess and a filling defect in the left renal vein, indicating RVT. These findings raised suspicion for pyelonephritis-induced renal thrombosis (Figure 2).

Based on these results, the patient underwent cystoscopic transurethral drainage of the prostatic abscess (TURP). The procedure was uneventful, and the patient was started on anticoagulation therapy with enoxaparin postoperatively. He initially recovered well and was able to pass urine. Before starting anticoagulation, blood was collected for a thrombophilia workup to rule out possible thrombophilic causes, and the results were all negative (Table 1). However, the urine and blood culture results were positive for MRSA, confirming the provisional diagnosis of pyelonephritis-induced renal thrombosis. Piperacillin/tazobactam was replaced with vancomycin, and enoxaparin was switched to rivaroxaban. The echocardiogram (ECHO) was performed and ruled out the presence of infective endocarditis.

On postoperative day 5, the Foley catheter was removed, and due to worsening renal function, vancomycin was switched to daptomycin. The patient improved and was subsequently discharged with antibiotics (co-trimoxazole/sulfamethoxazole for three weeks) and oral anticoagulation therapy (rivaroxaban, 15 mg twice daily for the first three weeks, followed by 20 mg once daily for an additional 69 days). He was scheduled for a follow-up in the outpatient clinic. The patient was evaluated during a follow-up visit 12 days after discharge and was found to be in excellent health. No complications or concerns were reported.

## Discussion

While rare, MRSA urinary tract infections (UTIs) can lead to serious complications such as RVT. RVT secondary to bacterial kidney infections is exceedingly uncommon but poses life-threatening risks if not promptly recognized and treated [4]. In this case, the patient presented with APN, renal abscesses, and MRSA bacteremia, which led to RVT, a rare but critical complication.

Thrombosis and inflammation are interconnected processes that contribute to "thrombo-inflammation," which can arise from severe infections, particularly during sepsis, or from noninfectious inflammatory states. In sepsis, dysregulation of the immune response can lead to disseminated intravascular coagulation (DIC), causing thrombotic complications throughout the microcirculation. Key players in this interplay include platelets, neutrophils, and monocytes. Platelets recognize bacteria via pattern recognition receptors, primarily Toll-like receptors (TLRs), and migrate within blood vessels to facilitate thrombus formation. They also bind to bacteria, activating neutrophils that produce neutrophil extracellular traps (NETs), which contain antibacterial components and contribute to thrombotic processes [5-7].

RVT development can be attributed to similar "thrombo-inflammatory" mechanisms in patients with APNs. While *Klebsiella pneumoniae *(KP) is typically associated with RVT due to endotoxin release and subsequent coagulation pathway activation, MRSA-induced RVT likely operates through a comparable inflammatory response. Although RVT is often linked to hypercoagulable states such as nephrotic syndrome, malignancy, and trauma, bacterial infections can also induce a prothrombotic state. Endotoxins from Gram-negative bacteria like KP activate tissue factors and inhibit fibrinolysis, contributing to thrombosis. In MRSA cases, the severe inflammatory response may similarly facilitate thrombus formation, illustrating how infections can lead to a procoagulant state and complications like RVT [8-10].

Clinically, RVT can be challenging to distinguish from pyelonephritis as both may present with fever, flank pain, and systemic signs of infection. Diagnosis requires a high index of suspicion, especially in patients with prolonged or atypical symptoms despite appropriate antibiotics. In this case, contrast-enhanced computed tomography (CT) identified the renal pyelonephritis with abscess and RVT. While CT angiography (CTA) is the preferred diagnostic method, magnetic resonance venography (MRV) and Doppler ultrasonography may be alternatives. Selective renal venography, once the gold standard for RVT diagnosis, has mainly been replaced by these less invasive techniques [8-9].

Treatment of RVT in bacterial pyelonephritis involves managing both the infection and the thrombus. This patient was started on low-molecular-weight heparin (LMWH), followed by rivaroxaban, a direct oral anticoagulant (DOAC). DOACs, like rivaroxaban, are increasingly used due to their ease of administration and comparable efficacy to traditional treatments with LMWH and vitamin K antagonists [10]. Although the optimal duration of anticoagulation remains debated, three months of therapy proved effective in these cases [11-12]. In addition to anticoagulation, treating the underlying infection is essential. The patient received a day course of intravenous antibiotics starting with vancomycin, daptomycin, and three weeks of oral co-trimoxazole. RVT in the context of pyelonephritis signifies disease severity and, if left untreated, can lead to complications such as inferior vena cava (IVC) extension or septic pulmonary embolism [13-14]. Although some cases of IVC extension may require surgical intervention or percutaneous thrombolysis, isolated RVT is typically managed conservatively with anticoagulation, as seen in this patient [15-17].

## Conclusions

Pyelonephritis can lead to serious complications, including the rare occurrence of RVT. Early diagnosis through imaging and cultures is essential, as RVT is driven by "thrombo-inflammation," an interaction between proinflammatory factors and the immune response that leads to thrombus formation. This case emphasizes the importance of early detection and treatment, as untreated RVT can result in severe outcomes like septic pulmonary embolism. With MRSA bacteremia, pyelonephritis, and abscess formation, our patient was successfully treated with anticoagulants (LMWH heparin followed by rivaroxaban) and targeted antibiotics. Further studies must determine the best approach for managing such rare complications.
